# Systems thinking methods: a worked example of supporting emergency medical services decision-makers to prioritize and contextually analyse potential interventions and their implementation

**DOI:** 10.1186/s12961-023-00982-y

**Published:** 2023-06-05

**Authors:** Cassandra Rehbock, Thomas Krafft, Anja Sommer, Carijn Beumer, Stefan K. Beckers, Stefan Thate, Jörn Kaminski, Alexandra Ziemann

**Affiliations:** 1 Department of Health, Ethics and Society, Care And Public Health Research Institute (CAPHRI), Faculty of Health, Medicine and Life Sciences, Maastricht University, Maastricht, 6229 ER The Netherlands; 2 Aachen Institute for Rescue Management and Public Safety, University Hospital RWTH Aachen and City of Aachen, 52068 Aachen, Germany; 3 City of Oldenburg - Fire Department, 26127 Oldenburg, Germany; 4 Oldenburg Research Network Emergency and Intensive Care Medicine (OFNI), Carl Von Ossietzky University, 26129 Oldenburg, Germany; 5 University Institute of Medical Informatics, University Hospital RWTH Aachen, 52057 Aachen, Germany; 6 Rettungsdienst Landkreis Oldenburg, 27793 Wildeshausen, Germany; 7 Department of Social and Policy Sciences, University of Bath, Bath, BA2 7AY United Kingdom

**Keywords:** Complexity, Decision support, Implementation

## Abstract

**Background:**

Systems thinking can be used as a participatory data collection and analysis tool to understand complex implementation contexts and their dynamics with interventions, and it can support the selection of tailored and effective implementation actions. A few previous studies have applied systems thinking methods, mainly causal loop diagrams, to prioritize interventions and to illustrate the respective implementation context. The present study aimed to explore how systems thinking methods can help decision-makers (1) understand locally specific causes and effects of a key issue and how they are interlinked, (2) identify the most relevant interventions and best fit in the system, and (3) prioritize potential interventions and contextually analyse the system and potential interventions.

**Methods:**

A case study approach was adopted in a regional emergency medical services (EMS) system in Germany. We applied systems thinking methods following three steps: (1) a causal loop diagram (CLD) with causes and effects (variables) of the key issue “rising EMS demand” was developed together with local decision-makers; (2) targeted interventions addressing the key issue were determined, and impacts and delays were used to identify best intervention variables to determine the system’s best fit for implementation; (3) based on steps 1 and 2, interventions were prioritized and, based on a pathway analysis related to a sample intervention, contextually analysed.

**Results:**

Thirty-seven variables were identified in the CLD. All of them, except for the key issue, relate to one of five interlinked subsystems. Five variables were identified as best fit for implementing three potential interventions. Based on predicted implementation difficulty and effect, as well as delays and best intervention variables, interventions were prioritized. The pathway analysis on the example of implementing a standardized structured triage tool highlighted certain contextual factors (e.g. relevant stakeholders, organizations), delays and related feedback loops (e.g. staff resource finiteness) that help decision-makers to tailor the implementation.

**Conclusions:**

Systems thinking methods can be used by local decision-makers to understand their local implementation context and assess its influence and dynamic connections to the implementation of a particular intervention, allowing them to develop tailored implementation and monitoring strategies.

**Supplementary Information:**

The online version contains supplementary material available at 10.1186/s12961-023-00982-y.

## Background

Prehospital emergency medical service (EMS) demand is continuously rising, causing EMS worldwide to struggle to provide the highest standard of patient care. The causes and effects of rising EMS demand are manifold and arise from different sectors and services, such as the organization of and access to primary care and out-of-hours healthcare services, as well as acute care services [1, 2]. A rapid review conducted by Turner et al. explored underlying factors related to the rise in demand for emergency and urgent care services in the English National Health Service (NHS) [3]. The study highlights that “one clear evidence gap identified [is] lack of studies that take a broader system approach to identifying, implementing and evaluating interventions to try to improve emergency and urgent care” (p. 93) [3]. The findings further indicate that a system perspective is necessary to evaluate specific interventions and to identify their target-oriented outcomes [3]. Insights on interventions related to the overall prehospital EMS system targeting the increasing demand could help in specifically targeting interventions and planning their implementation according to a certain setting. The need for a broader system approach is calling for an alternative method of analysing demand rise and supporting decision-makers in planning the implementation of interventions.

Systems thinking attempts to capture the system’s overall complexity, allowing the analysis of future possible scenarios [4]. As systems form a continuum of connected and embedded systems and subsystems, actors and elements, with superfluous and dynamic boundaries [4, 5], a change in one subsystem can have an impact on others, and vice versa [6]. This creates interdependencies and dynamics which can make any system increasingly complex [4, 5]. Similar features can be recognized in prehospital EMS, due to its three main sectors—ambulatory care/outpatient services, hospital care and ambulance services. Changes occurring in one sector influence the others and subsequently the overall emergency and urgent care system [7]. Some studies have used systems thinking to illustrate certain aspects of the overall system [8, 9], such as the issue of emergency department (ED) crowding related to policy implementations [8], but to our knowledge not from a wider perspective [3] on the general prehospital setting with its related subsystems.

Systems thinking can help to understand complex implementation contexts and their interactions with interventions, and selecting tailored and effective implementation actions [10]. In comparison to implementation efforts guided by predominant models or frameworks (e.g. in the form of a checklist) to determine contextual factors influencing implementation [11], systems thinking methods uncover causality and interactions from a whole system perspective [12, 13]. A review by Nilsen highlights that determinant frameworks imply rather linear relationships between factors and fail to address causalities [11]. Thus, their nature does not allow for the details needed to support stakeholders in the implementation of interventions in complex local contexts [11]. In addition, systems thinking methods illustrate in what way the dynamic nature of interventions influences a current state [12]. Applying systems thinking methods could allow for a much more tailored approach to identify interventions and plan their implementation.

Existing literature shows that the construction of causal loop diagrams (CLDs) is a frequently used and convenient systems thinking method to demonstrate all involved variables and their interconnections relating to a key issue across various sectors [14, 15]. Developing CLDs is one of the more frequently used mixed methods in the health setting [13]. A qualitative modelling process can offer insights into the dynamics of a complex system by illustrating delays between causes and effects and the resulting feedback structures emerging from interconnections between variables [12]. Powell and Mustafee, for example, report that qualitative system dynamics, compared to simulation-based analyses, help managers to structurally analyse and determine beneficial interventions based on the specific system dynamics [16]. Literature has shown the usefulness of CLDs to illustrate contextual factors to aid decision-making processes [17] and to support the implementation of interventions [18] in settings other than prehospital EMS.

Therefore, this study aimed to explore systems thinking methods using the output of a developed CLD manuscript to support decision-makers in preparing the implementation of interventions to tackle one key issue—rising EMS demand—in a worked example using a regional case study. The underlying objectives were thus threefold: (1) to understand locally specific causes and effects of the rise in EMS demand and how they are interlinked, (2) to identify the most relevant interventions and best fit in the system, and (3) to prioritize potential interventions and contextually analyse the system and potential interventions. The output gathered can assist the planning and tailoring of the implementation of interventions, and highlights the need for further systems thinking research in prehospital EMS in the future.

## Methods

This study combines three systems thinking methods steps in a case study of a regional EMS system in Germany. According to the legal framework for medical scientific research by the Dutch Central Committee on Research Involving Human Subjects, ethical approval was not needed.

### Case study

The case study is set in a regional EMS system in Oldenburg, Lower Saxony, Germany. In 2012, six dispatch centres, covering each of the six ambulance service areas in this region, were merged into one central dispatch centre situated in the city of Oldenburg (*Großleitstelle Oldenburger Land*) [19]. This forms a rather complex and unique EMS system. Recognizing differences in expertise and knowledge while also valuing the interdisciplinarity represented in the group is essential; thus, joint decision-making between the different stakeholders has become even more crucial. The dispatch centre covers a population of more than 735 000 citizens and a service area of around 4200 km^2^ [20].

Rising EMS demand, as identified by the stakeholders involved in this study, is an ongoing issue with high relevance for the EMS system in Oldenburg. Increasing numbers of nonemergency cases that do not require prehospital EMS to transport the patient to a healthcare facility pose a challenge and high burden on the regional services. According to the City of Oldenburg (fire brigade), cases in which outpatient care was performed by ambulance personnel rose in 2017, 2018 and 2019 from 6.120 to 7.199 and then up to 7.481 cases in the overall service area of EMS Oldenburg [21]. This includes cases where patients were examined and, if appropriate, cared for on-scene by EMS personnel but where transport to a healthcare facility was ultimately not necessary. Similarly, an increase in cases was observed where either the response was cancelled or no relevant patient was encountered by ambulance personnel on-scene [21].

### Systems thinking methods

We combined three systems thinking methods: (1) developing a CLD to illustrate system complexity and intersectorality related to rising EMS demand [12, 15], (2) determining targeted interventions tackling demand rise and identifying the best intervention variables (BIV) to analyse the interplay between impacts and time delays of interventions and to identify the best fit to implement an intervention in the system [22, 23], and (3) prioritizing potential interventions and contextually analysing their dependencies and consequences based on pathway analyses [24].

#### CLD

A first draft of the CLD was constructed during a 2-day workshop, which took place at the dispatch centre in Oldenburg, Germany. Twelve decision-makers of the regional ambulance services (senior management), the dispatch centre (senior management) in Oldenburg and the statutory health insurance agencies of Lower Saxony, Germany (division management, representatives/advisors) took part in the workshop. A core group of researchers (facilitators) organized and facilitated the discussions. Two facilitators (AZ, TK) moderated the joint session based on a previously drafted script, and two others (CR, KW) took notes to document the CLD design processes [25]. Rising EMS demand was explored, and decision-makers were asked to collect its potential causes and effects (variables). Variables and their connections to the key issue and other variables were firstly collected individually and then discussed jointly. The CLD was designed using Vensim PLE software (Personal Learning Edition, version 8.1.0) [26].

The relationships (causal links) between each of the identified variables in terms of their effect (polarity) on one another were determined and represented in the diagram (represented by arrows). Negative polarity constitutes an inversely proportional effect, meaning that a rise in variable A causes a decline in variable B and vice versa, whereas positive polarity represents a proportional relationship, meaning that a rise in variable A causes a rise in variable B and vice versa [12, 15].

Delays in effect between variables are also captured [12, 15], as defined by two of the workshop participants (senior management): (1) no delay (immediate effect), (2) short delay (up to 1 year) or (3) long delay (> 1 year). This step was undertaken after the variables and connections in the CLD were agreed on by all participants. A final CLD validation, including the identified relationships and delays between variables, was conducted collaboratively with these two stakeholders, and this was shared with all participants afterwards.

Feedback loops give more detail to the relationship between certain variables in a CLD. Examining the interconnections between variables may allow us to identify relationships that are circular and start and end with the same variables. They represent the dynamic within the system and depict variables influencing each other in a closed cycle, starting and ending at the same variable. The greater the number of feedback loops in a system, the more independent and less reactive to external variables the system is. At the same time, the greater the number of variables, and thus relationships, involved in a feedback loop, the longer the effects might take to occur [12]. Two kinds of feedback loops exist, reinforcing and balancing loops [12]. Each has different features and effects on a system, which are further outlined in the results section.

The relationships and delays between variables can help decision-makers to tailor actions according to the overall local context. Identifying and understanding feedback loops can support the identification of sources, either related to resistance and stability (balancing loops) or related to virtuous and vicious growth (reinforcing loops). The CLD can help decision-makers to visualize and understand a rather specific local problem, as well as any locally specific underlying multicausal aspects of rising EMS demand, thereby allowing for the selection of targeted interventions and a tailored plan for implementation to avoid failure.

#### Interventions and BIVs

During the workshop, participants were asked to identify possible interventions for tackling rising EMS demand. These were categorized by the workshop participants and illustrated in a matrix according to their effect (small to large) on rising demand and their expected implementation process in the EMS system (easy to difficult). Decision-makers based these decisions on experiences, chance of successful implementation in the regional setting and potential organizational resistance (e.g. political or staff-related). Based on this, three interventions were selected by the participants and incorporated into the CLD as external variables. “External” here indicates that they have an effect on the variables in the system but are not affected by the variables themselves. The stakeholders discussed the interventions’ connections to other variables in the CLD and how they would impact the key issue. This process was based on consensual decision-making among all participants.

BIVs are variables in the CLD that indicate the best fit for an intervention in a system, as their number of causal links in the system represents considerable change [22, 23]. To determine BIVs, two matrices, the cross-impact matrix (CIM) and the cross-time matrix (CTM) [22, 23], were created based on the workshop results. A CIM indicates which variables have a strong impact on the system—the higher the active sum (AS), the more outgoing flows and the higher the impact on the system; and the higher the passive sum (PS), the more incoming flows and the higher the system’s impacts on the respective variable. The active and passive sums are based on the previously identified relationships (causal links) between cause and effect variables [22, 23]. By adding the active and passive sums together, we obtain the respective cross-linking degree per variable: the higher the total sum, the more crucial the variable for the system, as it means that a variable is highly interconnected within the system structure (this means that the variable has many outgoing and incoming causal links to other variables) [23]. The CTM demonstrates the delay produced and received within the CLD. Produced delay (PD) is the average time an effect needs to reach a subsequent variable from variable A, representing the delay caused to the whole system, whereas received delay (RD) is the average time for an impulse to reach variable A. CTMs are based on the previously identified delays within the CLD [22, 23].

Eventually, the AS and PD values are combined, and variables that represent the best fit for an intervention are identified. BIVs generally have a higher AS, meaning many outgoing causal links and thus a strong effect on the system—and a lower PD and thus a quickly resulting change reaction [22, 23]. These variables need to be controllable for stakeholders to be able to regulate them [23]. While the delays and relationships within the CLD were determined by the participant group, the BIV determination was conducted by the research team subsequent to the workshop.

#### Intervention analysis

This analysis entailed a targeted, step-by-step analysis of an intervention in the system. By analysing sample pathways in the CLD, we aimed to demonstrate how the system might hypothetically react when implementing an intervention. The goal was to enable decision-makers to visualize how an intervention would interact with the system [24].

A two-sub-step approach was chosen: (1) decision-making to prioritize interventions based on previously identified BIVs and delays, and (2) context assessment based on the prioritized intervention, its related paths and its feedback loops (pathway analysis).

##### Prioritization of interventions

Firstly, we determined how many BIVs each intervention was connected to directly. This already indicated how quickly (based on PD) and to what extent (based on AS) change would happen in the system when implementing these interventions. However, BIVs do not indicate the impact on the key issue “rising EMS demand”. By analysing the direct delay between the interventions and the key issue, decision-makers can prioritize potential interventions and their implementation.

##### Pathway analysis

Secondly, we performed a contextual analysis using a sample intervention by analysing all direct connections between the interventions and variables (paths). We focused on explaining the relationships between variables and illustrated the impact of the involved feedback loops. We identified contextual factors following the categorization described by Damschroder et al. [27]. This context assessment can be used to determine locally specific implementation determinants as input to support decision-makers in tailoring implementation planning and the evaluation of a specific intervention.

## Results

### The CLD

**Fig. 1:**
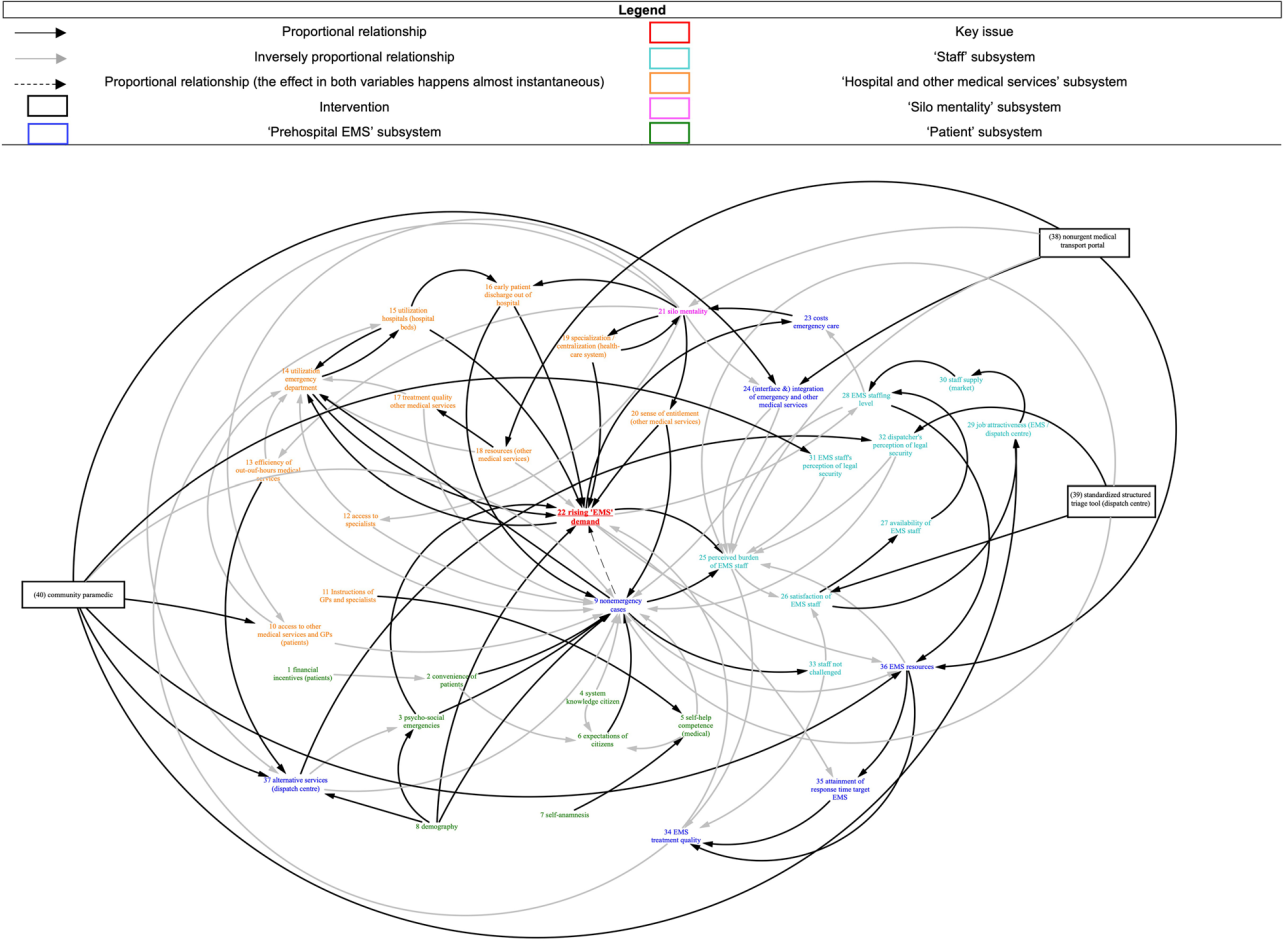
The overall causal loop diagram (CLD)

The final CLD is illustrated in Fig. 1. Thirty-seven variables were identified, and all of them, except for the key issue, were grouped in five thematic clusters (subsystems), as interlinked demand rise causes/consequences in the EMS system in the Oldenburg region. The subsystems illustrate the contextual factors involved and consist of (1) “hospital and other medical services”, (2) “patient”, (3) “staff”, (4) “prehospital EMS” and (5) “silo mentality”. Detailed subsystem explanations and variable definitions are given in Additional files 1 and 2, respectively.

An overview of the relationships between each variable illustrated in the CLD can be found in Fig. 1 and Additional file 3. Relationships between variables indicate the effect one variable has on another by means of a causal link. Delays between each variable are also summarized in Additional file 4. Delay distinguishes between those changes that happen immediately and need to be monitored first and those that happen at a later time and need to be monitored later.

Delays are not captured in the full CLD for illustrative purposes. Furthermore, we chose to illustrate selected feedback loops in an extract of the overall CLD only, to maintain a clear and useful level of visualization within the full CLD.

### Interventions and BIVs

Based on the intersectoral input gathered from the overall CLD diagram, a total of 13 interventions were identified. These varied from easily implementable to difficult and ranged from high to low effect. Three of these 13 were chosen (see Additional file 5) and incorporated into the CLD and are further described in Table 1. These three interventions are assumed to have a high effect, though with different degrees (intervention B having the highest), to counteract rising EMS demand. For two of those interventions, their implementation is presumed to be rather easy (A, C in Table 1), whereas the other one is assumed to be more difficult to implement (B in Table 1). Each intervention attaches to multiple variables in the CLD and across various subsystems, meaning that they will impact several areas in the overall system structure involving differing contextual factors.

**Table 1:** Possible interventions

Regional considerations				Decision-making and prioritization					
Possible interventions: their implementation and effect				Best intervention variables (BIVs)					
Intervention	Description and impact on rising EMS demand	Predicted implementation difficulty^a^	Predicted effect^a^	Directly connected BIVs		Active sum*	Produced delay**	Connection to key issue	Delay to key issue
A. Nonurgent medical transport portal	This electronic portal would be used to schedule (nonemergency) patient transports. Hospitals and dispatch centres would have access to the overview to schedule transport. This leads to improved discharge management due to simplified processes and more timely allocation of transport. This could enhance resource allocation and planning and thus counteract rising EMS demand.			36 EMS resources		3.0	1.0	38 ➔ **36 ➔ n ➔ 22**	++
B. Standardized structured triage tool (dispatch centre)	A standardized structured triage tool will allow for more standardized and structured dispatching processes. Patients could be directly referred to the right (emergency) medical service. The appropriate urgency level and resources could be dispatched. This would decrease unnecessary transport and counteract rising EMS demand.			9 Nonemergency cases		5.0	1.4	39 ➔** 9 ➔ 22**	+
C. Community paramedic	A community paramedic is most commonly dispatched in cases where it is clear that it is not an emergency, but the situation on-site and the kind of help needed is rather unclear or low-acuity. The community paramedic should be able to refer the patient to a hospital or other (medical) services. This would thus counteract rising EMS demand.			9 Nonemergency cases		5.0	1,4	40 ➔ **9 ➔ 22**	+
				36 EMS resources		3.0	1.0	40 ➔ **36 ➔ n ➔ 22**	++
				37 Alternative services (dispatch centre)		3.0	1.3	40 ➔** 37 ➔ 9 ➔ 22**	+^b^

^a^Predicted by the workshop participants;  easy,  rather easy,  very difficult,  moderately high effect,  rather high effect,  high effect; * min. 1.0, max. 8.0 active sum; ** min. 1.0, max. 3.5 produced delay; + = no delay (immediate effect); ++ = delay occurs; n = continuation of path over various variables

^b^Overall delay (the changes happen one after the other)

Based on the CIM, “silo mentality” shows the highest AS value. This indicates that it has the highest impact on the overall system among the variables. The highest passive sum is reported for “nonemergency cases”. This indicates that, compared to the others, it is impacted to a larger extent by the overall system. The highest degree of cross-linking between the active and passive sum values is noted for “nonemergency cases”. This indicates that it has a fundamental value in the overall system, due to its extensive interconnectedness compared to the other variables in the CLD.

Within the CTM, the highest PD is shown for “self-anamnesis”, “demography”, “specialization/centralization (healthcare system)”, “sense of entitlement (other medical services)”, “silo mentality”, “costs emergency care”, “satisfaction of EMS staff” and “job attractiveness (EMS/dispatch centre)”. This indicates that these variables generally generate slower effects throughout the system than other variables. On the other hand, the highest RD is reported for “access to other medical services and GPs (patients)” (general practitioners), “access to specialists”, “efficiency of out-of-hours medical services”, “specialization/centralization (healthcare system)”, “sense of entitlement (other medical services)”, “silo mentality”, “(interface &) integration of emergency and other medical services”, “availability of EMS staff”, “job attractiveness (EMS/dispatch centre)” and “staff supply (market)”. That means that these variables receive or thus respond to effect in a slower manner. More detailed results per variable can be found in Additional file 3 (CIM) and Additional file 4 (CTM).

Based on the values of PD and AS from the two matrices, the following five BIVs were identified (see Additional file 6, Fig. S1): “9—nonemergency cases”, “13—efficiency of out-of-hours medical services”, “15—utilization of hospitals (hospital beds)”, “36—EMS resources” and “37—alternative services (dispatch centre)”. All BIVs are controllable by decision-makers, as they lie within their decision-making sphere. Variables in quadrant II are also suitable for intervention, if a delay is manageable or possibly even required (slow implementation process). All three interventions directly target BIVs (Table 1); when introducing change (intervention) into the system, it might have a relatively high and quick impact on those variables.

### Intervention analysis

#### Prioritization of interventions

The intervention “community paramedic” directly connects to three BIVs, as illustrated in Table 1. BIVs indicate that changes initiated by the intervention would happen rather quickly and with a higher impact in those variables. Distinguishing between the three BIVs, a change in “nonemergency cases” has a higher impact on the overall system (AS) though with slightly greater PD than the BIVs “EMS resources” and “alternative services”. In comparison, “nonurgent medical transport portal” and “standardized structured triage tool” (hereafter, “triage tool”) both connect to one BIV only.

“Triage tool” and “community paramedic” both directly connect to “nonemergency cases”, while “nonurgent medical transport portal” connects to a BIV that does not directly impact the key issue. The overall delay here is larger, due to various interconnections between several variables. Due to their overall quicker impact on the central issue, “community paramedic” and “triage tool” should be prioritized over “nonurgent medical transport portal”.

#### Pathway analysis

**Fig. 2:**
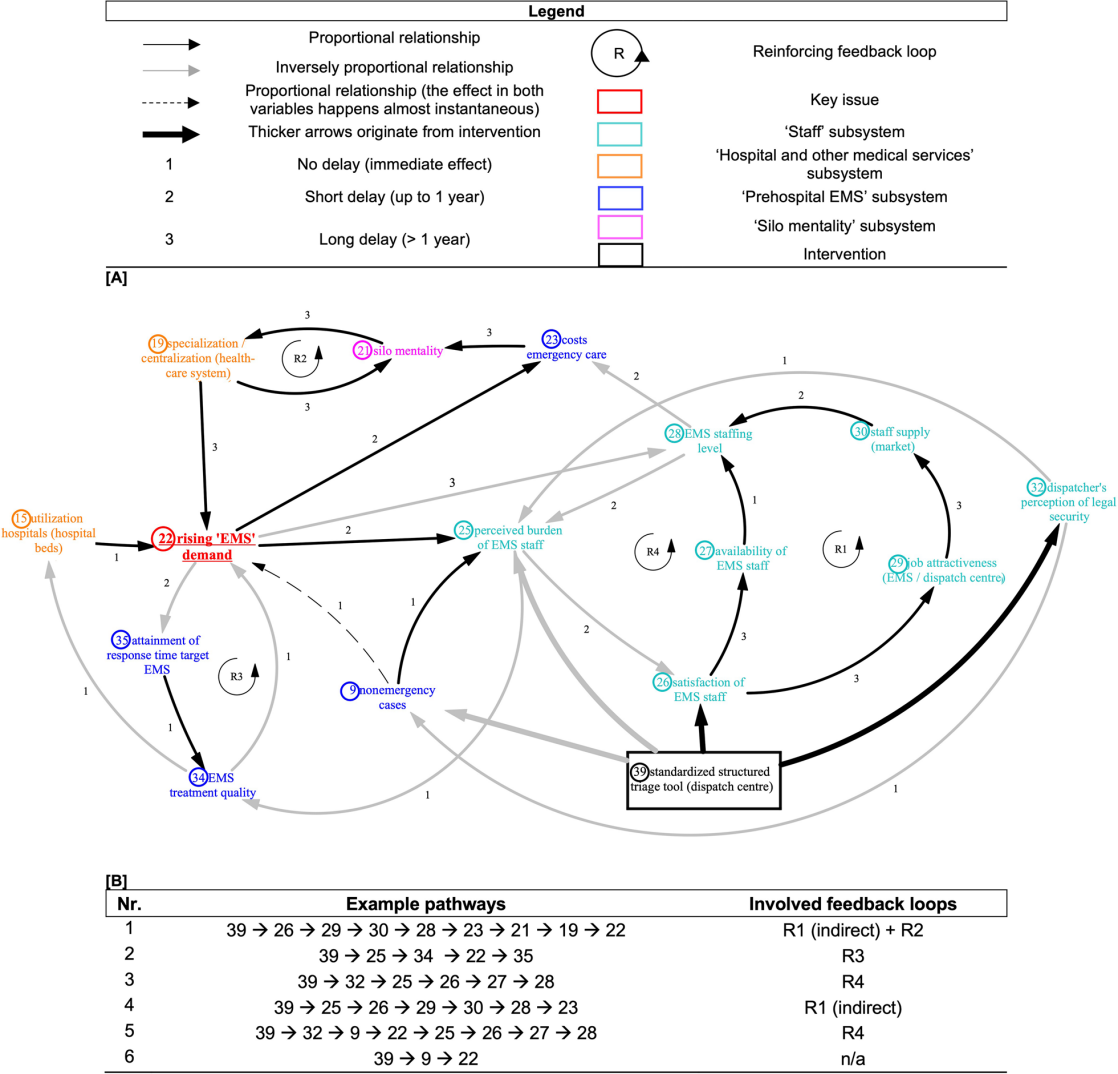
Causal loop diagram extract of the “standardized structured triage tool”, illustrating delays and feedback loops

Using pathway analysis, we illustrate how the sample innovation triage tool would fit into the system and we highlight the connections to the implementation context. In Fig. 2A we can identify groups of contextual determinants and stakeholders that relate to the implementation of the triage tool. There is a group of organizational or “inner” contextual factors related to organizational characteristics or the organizational culture (e.g. staff satisfaction) of the EMS. Further, there is a group of external contextual factors related more to structural components of the prehospital EMS system beyond the organization (e.g. silo mentality and other organizations [hospitals]). Another group of contextual factors can be identified that is related to individual characteristics of EMS staff in the dispatch centre and the ambulance service. Comparing the pathway for the triage tool to the overall CLD (see Fig. 1) demonstrates that the stakeholder groups of patients or citizens, as well as primary care or specialized ambulatory care providers, play a lesser role in the implementation of the triage tool. The pathway analysis helps to identify those local contextual factors and stakeholder groups that play a role in the implementation of the triage tool. Decision-makers could select implementation and engagement strategies targeting these contextual factors and stakeholder groups with priority.

Looking at the delays and feedback loops that are part of the pathway can be used for further prioritization in the implementation process. Delays indicate that changes occur later on in the implementation process, which can indicate later required action by decision-makers. A reinforcing feedback loop demonstrates variables influencing each other in a growing or declining action. A balancing loop illustrates that variables influence each other with a neutralizing or self-regulating effect. While balancing loops can build up either resistance or stability within a system and aim at a goal, reinforcing loops can develop to be virtuous (positive effect/goal-oriented behaviour) or vicious (negative effect) by developing exponentially in the direction of change (“growth” or “decline”) [12, 28]. To illustrate this, we depict a reinforcing loop in one example. Path 3 in Fig. 2B shows that a structured and standardized triage process will improve the dispatchers’ legal security perception. This points to this being a lever or key focus point in the implementation process. Thinking about the implementation strategy, decision-makers could choose to focus on this topic in the training and dissemination activities targeting EMS staff. A structured standardized triage tool subsequently improves their perceived burden and ultimately increases satisfaction. Over a longer period, staff availability will increase and will immediately result in improved staffing levels or increase staff retention. This means that the effects of improved staff satisfaction will not be visible immediately. However, even though staff satisfaction may increase, staff availability is finite; an endless number of staff cannot be employed or paid. The rising availability of staff may also decrease again over time, which increases the level of scarcity of employable staff. Decision-makers can use this information, observing the fluctuations in staff availability and making informed decisions regarding staff employments, while monitoring the intervention’s effectiveness.

The pathway analysis also helps to reveal certain parts of the system that could be monitored as part of the evaluation of the implementation effectiveness, particularly the variables around EMS’s attainment of the response time target, EMS treatment quality and number of nonemergency cases.

## Discussion

By using a three-step systems thinking approach, we were able to conceptualize a system-based CLD around a particular problem (rising EMS demand) in a specific local context, identify and prioritize interventions and their best fit within the system, and assess the diagram to identify specific implementation determinants and stakeholder groups to support decision-makers in tailoring their implementation strategy. The systems thinking approach used allows us to prioritize the implementation of interventions and gives decision-makers insights into which aspects to address early on and which at a later time. They can subsequently use the CLD’s output to inform the implementation process, as well as monitor and evaluate its impact and progress.

We were able to map the underlying contextual factors of a key issue involved in a regional setting, such as stakeholders and inner and outer contextual factors. A study by Sarkies et al. demonstrates the usefulness of using CLDs to illustrate contextual factors [17]. The study gathered determinants related to the implementation of an intervention based on the Consolidated Framework for Implementation Research (CFIR) and demonstrated the relationships between them [17]. We used the CLD to gather and demonstrate underlying factors and their interrelationships, as well as any potential delays or feedback loops involved, and highlighted the usefulness of these findings for tailored decision-making in implementation.

Our study also proposes systems thinking methods for decision-making and the prioritization of interventions. An example of a study that used a CLD as the basis to explore possible interventions and to support their implementation is that by Kang et al. [18]. The authors applied three steps to prospectively plan suitable interventions to improve treatment of chronic kidney disease. Based on the literature and stakeholder interviews, the researchers constructed a CLD and used it as input for implementation planning of interventions. This resulted in a prioritization of interventions for implementation [18]. In the present study, we integrated further analyses and insights, including the best fit for intervention (i.e. BIVs) and pathway analysis. Schoenenberger et al. used a path analysis to identify intended or unintended consequences of potential policy reforms targeting ED crowding, by identifying relative impacts and delays between impact and effect variables [8]. Similarly, we used the CLD as a basis for the pathway analysis, utilizing the information on the relationship and delay between variables. We further highlighted the contextual factors involved, best fit for intervention and feedback mechanisms. These support the implementation planning and tailoring of the implementation process to the needs of the setting. The combination of BIVs, predicted implementation difficulty and effect, and the intervention’s connection to the key issue and delay to the key issue give decision-makers a basis to prioritize interventions and tailor their implementation.

Previous studies have illustrated their CLDs based on literature reviews or interviews [18, 29, 30]. Our CLD was created directly together with decision-makers by collaboratively identifying the variables and drawing up the system map. This was done to illustrate the specific local setting and possible effects of potential interventions but also to support their decision-making. This can be seen as a particular strength of this study. Decision-makers are directly involved in mapping the system, which enhances their understanding of underlying factors and allows them to critically assess the situation to formulate targeted interventions. Additionally, earlier studies have used the CLD as a method to visually demonstrate their study output based on the gathered findings [8, 17, 30]. We have used the method to collect and analyse data provided by the decision-makers and using it to inform tailored implementation planning.

### Novel contribution and outlook

Systems thinking in the health setting helps planners and stakeholders understand underlying complexities and interdependencies between subsystems. It promotes an understanding of potential outcomes of causes and effects and that implementing interventions at a small scale does not guarantee success at a large scale [31]. It also strengthens information exchange amongst the various involved stakeholders in health systems. Encouraging collaborative action and problem-solving promotes health system strengthening, particularly by identifying fitting interventions [24].

This systems thinking approach, illustrating the key issue of rising EMS demand in a local setting, is a new approach in the EMS setting. While systems thinking approaches in other settings [23], healthcare settings [32, 33] or specific contexts of EMS [8] have been researched, the system-wide approach is lacking in prehospital EMS [3]. The (successful) implementation of change in EMS requires the consideration and understanding of rising demand across all involved subsystems. Otherwise, interventions might seem to be failing when they are not, when not observed from a broader system point of view. Turner et al. recommended gaining a wider perspective in this area to be able “to encompass the wider system issues” (p. 93) [3].

This study is one example of how to gain this understanding by illustrating the causes and effects of rising EMS demand from a broad system point of view and helping decision-makers to undertake informed decisions in tailoring the implementation of future interventions.

The results of this study give decision-makers a basis to understand the interlinks in the broader EMS system, to prioritize interventions and to develop tailored implementation based on the system’s need. This approach may be useful in other healthcare settings to inform decision-makers prior to the implementation of novel interventions. In particular, prehospital EMS—a fast-working, time-sensitive and crucial part of any healthcare system—requires tailor-made implementation of interventions. Thus, further research using systems thinking approaches like this would benefit the field of prehospital EMS. Additional research into making specific implementation recommendations based on illustrating implementation determinants would be a necessary next step.

## Limitations

Our study has a few methodological limitations. The involvement of a more diverse group of stakeholders could have resulted in more input for the CLD and the analytical steps. This includes for example patients, patient representatives or practitioners from other medical services. Their input may have led to additional variables that patients or e.g. GPs/hospital physicians may have considered relevant with respect to rising EMS demand. Their perspective could lead to a better overall understanding of the dynamics based on a broader audience. However, this was not within the scope of this study. We focused on decision-makers and thus only included them in this study.

While the expertise of the two participants classifying the delays was fitting for the purpose of highlighting the time factors involved, a more diverse group of people could have given even more insights and may have led to slightly differing results.

Although we were able to identify contextual factors that could be used to tailor implementation planning, we did not specifically focus on illustrating implementation determinants in this study. The aim of the study was to illustrate and describe a certain key issue in a regional setting, which allowed us to gather any related and relevant variables from the decision-makers’ perspective. Based on the study’s findings, we cannot make specific implementation recommendations but can help to tailor the planning.

To our knowledge, no test or validation tool of the construction of a CLD currently exists. A tool might indicate whether certain variables were missed or that the process was lacking certain details. However, the absence of such a tool is mainly based on the fact that the involved variables and their illustrated relationships are often grounded on knowledge or experiences. No exhaustive list, or right or wrong answers exist. Instead, the CLD depicts key variables and focuses on relevant topics, determinants or stakeholder groups.

## Conclusion

Systems thinking methods can be used by local decision-makers to understand their local implementation context and assess its influence and dynamic connections to the implementation of particular interventions. The three-step approach gathered useful findings, while actively involving stakeholders in the data collection phase. The gathered results give decision-makers insights into which aspects to address early on and which at a later time. This allows them to develop tailored implementation and monitoring strategies for interventions.

## Supplementary Information

**Additional file 1. **The causal loop diagram.

**Additional file 2. **Variable descriptions and subsystems.

**Additional file 3. **Cross-impact matrix.

**Additional file 4. **Cross-time matrix.

**Additional file 5. **Identification of interventions, their implementation process and their effects.

**Additional file 6. **The best intervention variables.

## Data Availability

All data generated or analysed during this study are included in this published article and its additional files.

## Abbreviations

AS: Active sum
BIV: Best intervention variable
CLD: Causal loop diagram
CFIR: Consolidated Framework for Implementation Research
CIM: Cross-impact matrix
CTM: Cross-time matrix
ED: Emergency department
EMS: Emergency medical services
GP: General practitioner
PD: Produced delay
