# Supplementary material for: Systems thinking methods: a worked example of supporting emergency medical services decision-makers to prioritize and contextually analyse potential interventions and their implementation
Source: Health Res Policy Syst. 2023 Jun 5;21:42. doi: 10.1186/s12961-023-00982-y (PMC10242989; doi:10.1186/s12961-023-00982-y)
Supplement: Supplementary file 1 — Additional file 1. The causal loop diagram. [file 12961_2023_982_MOESM1_ESM.pdf]

Additional file 1. Sub-system descriptions

Figure 1 in the main text illustrates the respective sub-systems, which are further explained here. Sub-system 1 'hospital and other medical services' (orange) includes variables related to the availability and access to the hospital setting, general practitioners (GP)/ specialists and out of hours services sectors. Any bottlenecks in this sub-system may result in citizens seeking help via prehospital EMS services directly, causing rise in demand.

Variables in sub-system 2 'patient' (green) indicate the patient's role in the system. A general understanding of the system, as well as self-help competence could contribute to allocating resources according to chief complaints. Lack of this can cause patients to seek help with EMS directly, as they may not be aware of alternative services. This would thus increase demand on EMS.

Staff satisfaction and availability are crucial factors for a functioning EMS system. Variables in sub-system 3 'staff' (bright blue) focus on stress, legal security, satisfaction and job attraction, which in turn determine adequate staffing and recruitment of dispatch center and/ or ambulance service personnel. Many variables in this sub-system are not directly connected to the variable rising EMS demand but are interconnected effects of the key issue and underlying sub-systems.

The 'prehospital EMS' (dark blue) variables in sub-system 4 consist of costs, access to other health services, treatment quality and adequate service coverage of the EMS system. A lack of collaboration (availability of out-of-hours (OOH) medical services or a technical connection) between EMS services and alternative services may cause an increase in EMS demand and more patients will be cared for and transported by EMS directly.

Sub-system 5 or 'silo mentality' (pink) consists of one variable only. Although the CLD represents the interconnections between the various sub-systems from a whole systems perspective, these underlying sectors often do not work together due to specialization of services (ED vs. GP services). Lack of collaboration and of a central point of access for patients can cause an increase in EMS demand, as services will not be able to navigate the patient jointly.
