# Supplementary material for: Systems thinking methods: a worked example of supporting emergency medical services decision-makers to prioritize and contextually analyse potential interventions and their implementation
Source: Health Res Policy Syst. 2023 Jun 5;21:42. doi: 10.1186/s12961-023-00982-y (PMC10242989; doi:10.1186/s12961-023-00982-y)
Supplement: Supplementary file 2 — Additional file 2. Variable descriptions and subsystems. [file 12961_2023_982_MOESM2_ESM.pdf]

Additional file 2

Table S1. Variable descriptions and sub-systems.

#	Sub-systems	Variables	Descriptions
1	*Patient*	Financial incentives (patients)	Own contribution to EMS (emergency medical services) costs by the citizen/ patient (examples: own risk, deductible).
2	*Patient*	Convenience of patients	The patients' perception that the access to the EMS system is comfortable, free of charge and easy.
3	*Patient*	Psycho-social emergencies	Patients with psychosocial problems (German abbreviation: PsychKG).
4	*Patient*	System knowledge citizen	The patients'/ citizens' knowledge of appropriateness of, availability of and access to medical services (direct connection to the information flow between doctor and patient).
5	*Patient*	Self-help competence (medical)	The possibility for the citizens to determine and treat medical concerns themselves.
6	*Patient*	Expectations of citizens	The citizens' claim of entitlement regarding EMS.
7	*Patient*	Self-anamnesis	An anamnesis performed by the citizens/ patients themselves.
8	*Patient*	Demography	Changes in the population numbers and their constellations within the EMS Oldenburg service area.
9	*Pre-hospital EMS*	Non-emergency cases	Responses that do not require any emergency care or service but are still dispatched and transported by EMS.
10	*Hospital and other medical services*	Access to other medical services and general practitioners (GPs) (patients)	The patients' availability and access to other medical services (excl. specialists).
11	*Hospital and other medical services*	Instructions of GPs and specialists	Sufficient briefing and dissemination of medical information to the patient by GPs and specialists.
12	*Hospital and other medical services*	Access to specialists	Availability and accessibility of specialists for the patients.
13	*Hospital and other medical services*	Efficiency of out-of-hours medical services	The efficiency of out-of-hours (OOH) medical services (examples: access, availability, completion of transferred tasks, quality of service).
14	*Hospital and other medical services*	Utilization emergency department	The used capacity of the emergency department.
15	*Hospital and other medical services*	Utilization hospitals (hospital beds)	The used capacity of beds in a hospital.

16	*Hospital and other medical services*	Early patient discharge out of hospital	The discharge of patients from the hospital before they have sufficiently recovered.
17	*Hospital and other medical services*	Treatment quality other medical services	The quality of care by other medical services (excl. dispatch center/ EMS).
18	*Hospital and other medical services*	Resources (other medical services)	Availability of material and staffing resources of other medical services (excl. dispatch center/ EMS).
19	*Hospital and other medical services*	Specialisation/ centralisation (health care system)	The concentration of services at certain, mainly superordinate service providers, such as the treatment of seriously injured persons in trauma centers that are only located at larger hospitals.
20	*Hospital and other medical services*	Sense of entitlement (other medical services)	The expectation of other medical services regarding the services provided by EMS.
21	*System dynamics*	Silo mentality	A thought-process with the sole focus on a respective sector/ institution; the opposite of systems thinking.
22	***Key issue***	Rising ‘EMS‘ demand	The demand of EMS in the service area of EMS Oldenburg.
23	*Pre-hospital EMS*	Costs emergency care	The total costs of emergency care.
24	*Pre-hospital EMS*	(Interface &) Integration of emergency and other medical services	The technical connection of the various medical services involved (i.e. prehospital EMS, emergency department, OOH services).
25	*Staff*	Perceived burden of EMS staff*	The perception of the EMS staff regarding the arising burdens due to their tasks and functions.
26	*Staff*	Satisfaction of EMS staff*	The satisfaction of EMS staff.
27	*Staff*	Availability of EMS staff*	On the one hand the loss of staff due to i.e. illness/ notice of termination or on the other hand the successful recruitment of staff.
28	*Staff*	EMS staffing level	Employed and available staff per service area or dispatch center.
29	*Staff*	Job attractiveness (EMS / dispatch center)	Attractiveness of the job descriptions in EMS.
30	*Staff*	Staff supply (market)	Availability of qualified EMS personnel on the market.
31	*Staff*	EMS staff’s perception of legal security	The EMS staff’s perceptions in the field on the possible legal consequences of their decisions. For example, when they decide to transport or treat and leave a patient.
32	*Staff*	Dispatcher’s perception of legal security	The dispatchers’ perception on the possible legal consequences of their decisions. For example, the choice to send a certain kind of response or

			none at all to a patient or the decision to refer a patient to another (medical) service.
33	*Staff*	Staff not challenged	The expectation of a service that is below the skills, abilities and knowledge of EMS personnel.
34	*Pre-hospital EMS*	EMS treatment quality	The quality of EMS.
35	*Pre-hospital EMS*	Attainment of response time target EMS	The attainment of the legally set response time interval in the service area (starting from the incoming call to the EMS arrival on scene).
36	*Pre-hospital EMS*	EMS resources	The availability of EMS resources to cover the demand. This includes material resources, such as vehicles but also staff resources (including temporary employment and long-term demand planning).
37	*Pre-hospital EMS*	Alternative services (dispatch center)	The possibility of the dispatch center to transfer a call/ case to the alternative social or medical services.

*EMS staff includes both ambulance and dispatch personnel.
