# Supplementary material for: Systems thinking methods: a worked example of supporting emergency medical services decision-makers to prioritize and contextually analyse potential interventions and their implementation
Source: Health Res Policy Syst. 2023 Jun 5;21:42. doi: 10.1186/s12961-023-00982-y (PMC10242989; doi:10.1186/s12961-023-00982-y)
Supplement: Supplementary file 3 — Additional file 3. Cross-impact matrix. [file 12961_2023_982_MOESM3_ESM.pdf]

S3 Table. Cross-impact matrix.

	CROSS-IMPACT MATRIX	1	2	3	4	5	6	7	8	9	10	11	12	13	14	15	16	17	18	19	20	21	22	23	24	25	26	27	28	29	30	31	32	33	34	35	36	37	AS
**1**	Financial incentives (patients)		-1																																				1
**2**	Convenience of patients						-1			1																													2
**3**	Psycho-social emergencies									1													1																2
**4**	System knowledge citizen						-1			-1																													2
**5**	Self-help competence (medical)						-1			-1																													2
**6**	Expectations of citizens									1																													1
**7**	Self-anamnesis					1																																	1
**8**	Demography			1						1													1															1	4
**9**	Non-emergency cases														1								1			1								1			-1		5
**10**	Access to other medical services and GPs (patients)									-1					-1																								2
**11**	Instructions of GPs and specialists					1																																	1
**12**	Access to specialists									-1					-1																								2
**13**	Efficiency of out-of-hours medical services									-1					-1																							1	3
**14**	Utilization emergency department															1							1																2
**15**	Utilization hospitals (hospital beds)														1		1						1																3
**16**	Early patient discharge out of hospital									1													1																2
**17**	Treatment quality other medical services									-1					-1																								2
**18**	Resources (other medical services)														-1			1					-1																3
**19**	Specilisation/ centralisation (health care system)																					1	1																2
**20**	Sense of entitlement (other medical services)									1													1																2
**21**	Silo mentality										-1		-1	-1			1			1	1				-1													-1	8
**22**	**Rising EMS demand**														1									1		1			-1							-1	-1		6
**23**	Costs emergency care																					1																	1
**24**	(Interface &) Integration of emergency and other medical services									-1																-1													2
**25**	Perceived burden of EMS staff																										-1								-1				2
**26**	Satisfaction of EMS staff																											1		1									2
**27**	Availability of EMS staff																												1										1
**28**	EMS staffing level																							-1		-1											1		3
**29**	Job attractiveness (EMS/ dispatch center)																														1								1
**30**	Staff supply (market)																												1										1
**31**	EMS staff's perception of legal security																									-1													1
**32**	Dispatchers's perception of legal security									-1																-1													2
**33**	Staff not challenged																										-1								-1				2
**34**	EMS treatment quality															-1							-1																2
**35**	Attainment of response time target EMS																																		1				1
**36**	EMS resources																									-1									1	1			3
**37**	Alternative services (dispatch center)			-1						-1																							1						3
	**PS**	0	1	2	0	2	3	0	0	15	1	0	1	1	8	2	2	1	0	1	1	2	10	2	1	7	2	1	3	1	1	0	1	1	4	2	3	3	
	**Degree of cross-linking (AS+PS)**	1	3	4	2	4	4	1	4	20	3	1	3	4	10	5	4	3	3	3	3	10	16	3	3	9	4	2	6	2	2	1	3	3	5	3	6	6	

<u>**Legend**</u>

Impact:

-1: Inversely proportional

Empty cell: No Influence/ no connection

+1: Proportional

AS = Active sum

PS = Passive sum

AS average	2,3	**SD**	1,5
PS average	2,3	**SD**	3,1
Average degree of cross-linking	4,6	**SD**	4,0
