# Supplementary material for: Systems thinking methods: a worked example of supporting emergency medical services decision-makers to prioritize and contextually analyse potential interventions and their implementation
Source: Health Res Policy Syst. 2023 Jun 5;21:42. doi: 10.1186/s12961-023-00982-y (PMC10242989; doi:10.1186/s12961-023-00982-y)
Supplement: Supplementary file 4 — Additional file 4. Cross-time matrix. [file 12961_2023_982_MOESM4_ESM.pdf]

S4 Table. Cross-time matrix

	CROSS-TIME MATRIX	1	2	3	4	5	6	7	8	9	10	11	12	13	14	15	16	17	18	19	20	21	22	23	24	25	26	27	28	29	30	31	32	33	34	35	36	37	PD
1	Financial incentives (patients)		1																																				1,0
2	Convenience of patients						3			1																													2,0
3	Psycho-social emergencies									1													1																1,0
4	System knowledge citizen						1			1																													1,0
5	Self-help competence (medical)						1			1																													1,0
6	Expectations of citizens									1																													1,0
7	Self-anamnesis					3																																	3,0
8	Demography			3						3													3															3	3,0
9	Non-emergency cases														1								1			1								2			2		1,4
10	Access to other medical services and GPs (patients)									1					1																								1,0
11	Instructions of GPs and specialists					2																																	2,0
12	Access to specialists									2					2																								2,0
13	Efficiency of out-of-hours medical services									1					1																							1	1,0
14	Utilization emergency department															1							1																1,0
15	Utilization hospitals (hospital beds)														1		1						1																1,0
16	Early patient discharge out of hospital									2													2																2,0
17	Treatment quality other medical services									2					2																								2,0
18	Resources (other medical services)														2			1					2																1,7
19	Specilisation/ centralisation (health care system)																					3	3																3,0
20	Sense of entitlement (other medical services)									3													3																3,0
21	Silo mentality										3		3	3			3			3	3				3													3	3,0
22	**Rising EMS demand**														1									2		2			3							2	1		1,8
23	Costs emergency care																					3																	3,0
24	(Interface &) Integration of emergency and other medical services									2																2													2,0
25	Perceived burden of EMS staff																										2								1				1,5
26	Satisfaction of EMS staff																											3		3									3,0
27	Availability of EMS staff																												1										1,0
28	EMS staffing level																							2		2											2		2,0
29	Job attractiveness (EMS/ dispatch center)																														3								3,0
30	Staff supply (market)																												2										2,0
31	EMS staff's perception of legal security																									1													1,0
32	Dispatchers's perception of legal security									1																1													1,0
33	Staff not challenged																										2								3				2,5
34	EMS treatment quality															1							1																1,0
35	Attainment of response time target EMS																																		1				1,0
36	EMS resources																									1									1	1			1,0
37	Alternative services (dispatch center)			1						1																							2						1,3
	RD		1,0	2,0		2,5	1,7			1,5	3,0		3,0	3,0	1,4	1,0	2,0	1,0		3,0	3,0	3,0	1,8	2,0	3,0	1,4	2,0	3,0	2,0	3,0	3,0		2,0	2,0	1,5	1,5	1,7	2,3	

Legend

Delay:

Empty: No effect (as there is no connection)

1: Immediate effect (no delay)

2: delay of up to 1 year (short delay)

3: delay of >1 year (long delay)

PD = Produced delay

RD = Received delay

Average RD	2,1	**SD**	0,7
Average PD	1,8	**SD**	0,8
