# Supplementary material for: Systems thinking methods: a worked example of supporting emergency medical services decision-makers to prioritize and contextually analyse potential interventions and their implementation
Source: Health Res Policy Syst. 2023 Jun 5;21:42. doi: 10.1186/s12961-023-00982-y (PMC10242989; doi:10.1186/s12961-023-00982-y)
Supplement: Supplementary file 5 — Additional file 5. Identification of interventions, their implementation process and their effects. [file 12961_2023_982_MOESM5_ESM.pdf]

Additional file 5. Identification of interventions, their implementation process and their effects

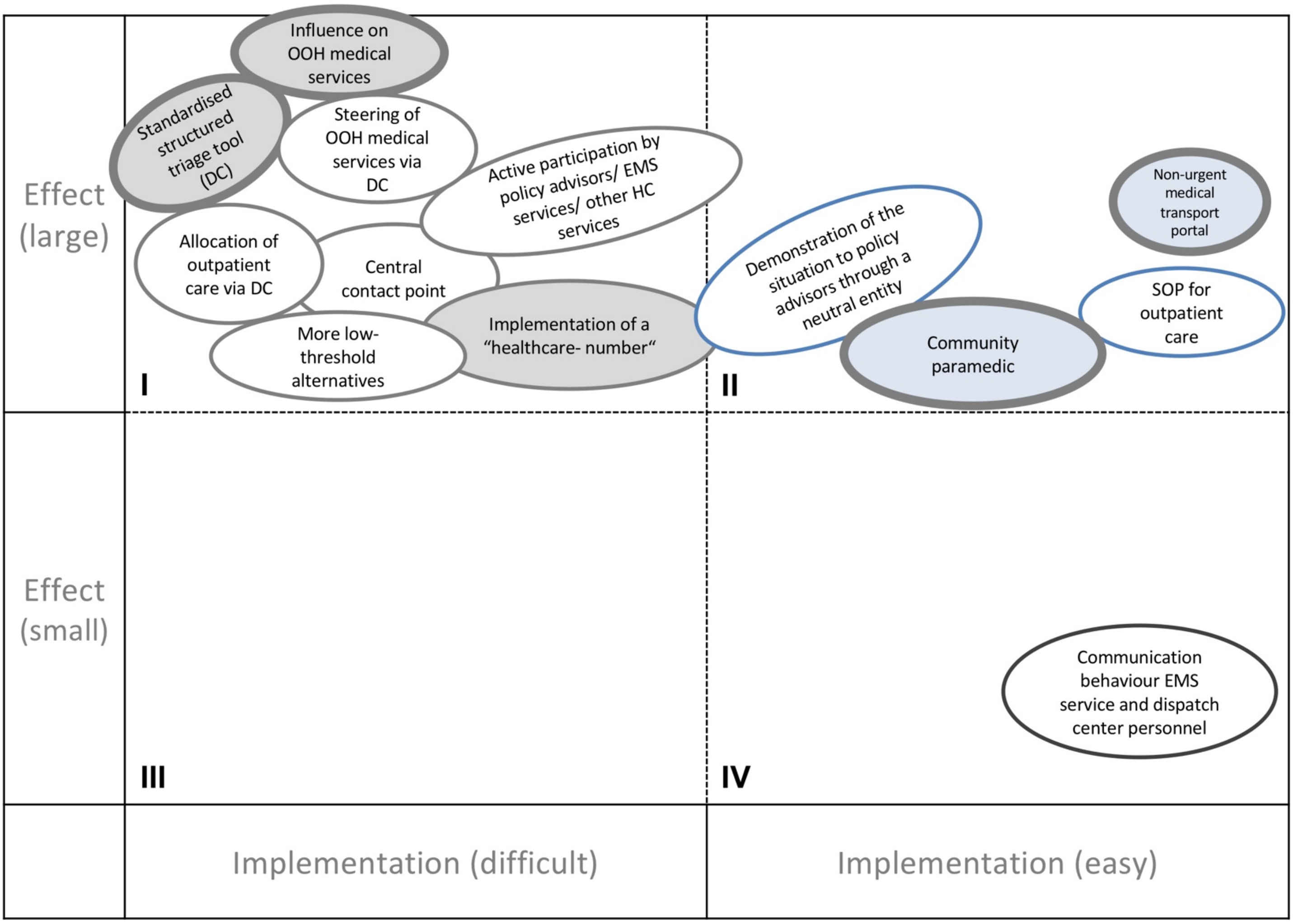

Figure S1. Effect and difficulty of implementation of proposed interventions. DC = Dispatch Center; EMS = Emergency Medical Services; HC = Healthcare OOH = Out-Of-Hours; SOP = Standard Operating Procedures

A total of 13 interventions were identified. The interventions were illustrated in a matrix according to effect and difficulty of implementation (Fig 1). A majority of interventions were placed in quadrant I by having a large effect on the central issue and being rather difficult to implement. The difficulty of implementation was assessed by means of the required influence on other sectors, such as politics or on other operational levels, such as national versus regional changes. Interventions in quadrant II represent changes with a large effect and a small effort to implement, which makes these interventions favorable. Though interventions in quadrant IV have a smaller effect, their implementation is least difficult, compared to the other interventions. The color-coded interventions were prioritized by

participants. The highlighted interventions (thick grey circle) were of biggest interest to the stakeholders, as they identified them to be implementable in their own EMS setting. Based on Figure 1 and considering regional aspects, three interventions were subsequently illustrated in the CLD: (1) ‘non-urgent medical transport portal’, (2) ‘community paramedic’ and (3) ‘standardized structured triage tool (dispatch center)’.
