# Supplementary material for: Systems thinking methods: a worked example of supporting emergency medical services decision-makers to prioritize and contextually analyse potential interventions and their implementation
Source: Health Res Policy Syst. 2023 Jun 5;21:42. doi: 10.1186/s12961-023-00982-y (PMC10242989; doi:10.1186/s12961-023-00982-y)
Supplement: Supplementary file 6 — Additional file 6. The best intervention variables. [file 12961_2023_982_MOESM6_ESM.pdf]

Additional file 6. The best intervention variables

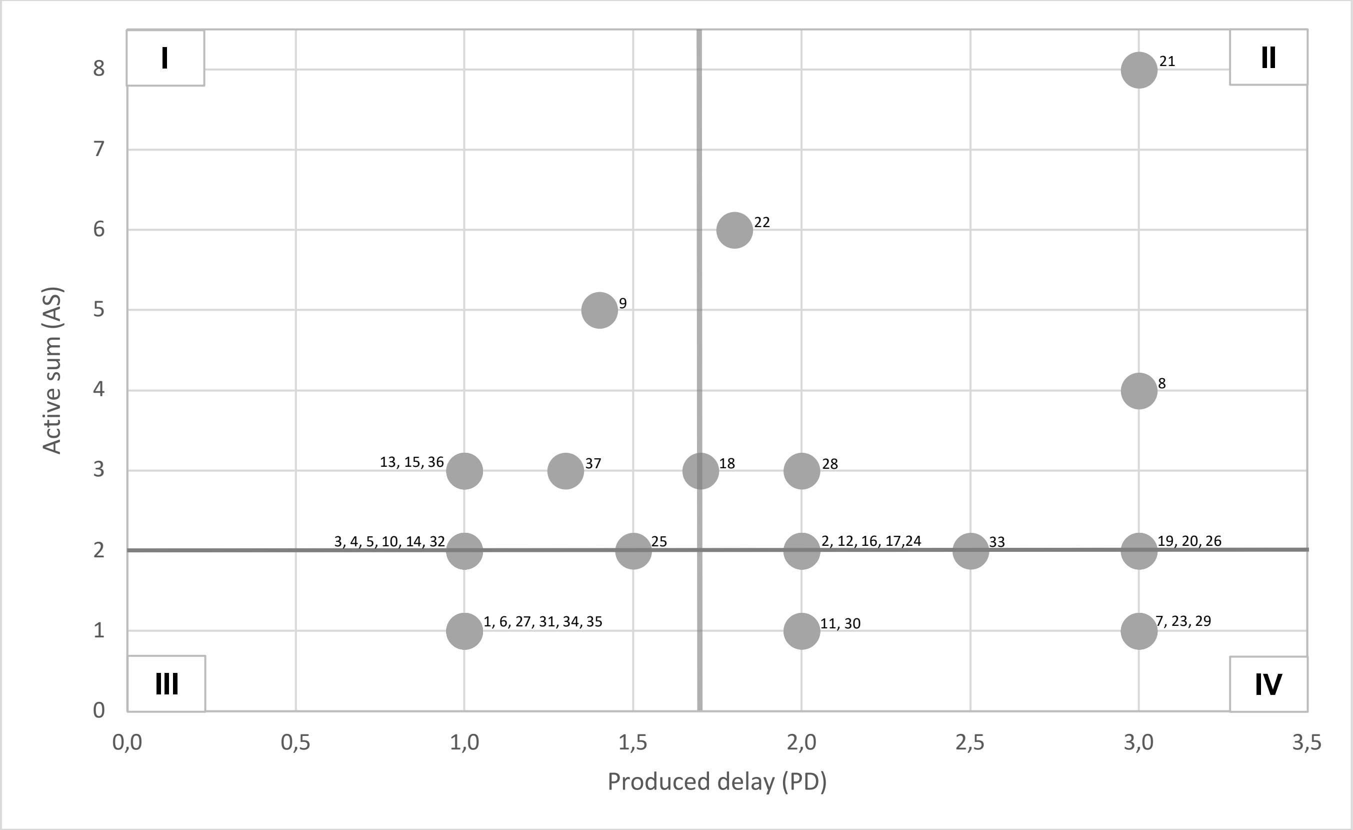

Fig. S1 The identification of best intervention variables. The corresponding variable names to the illustrated numbers can be cross-checked with Supplement 1 . *BIVs are represented in quadrant I, as these variables generally have a higher active sum and a lower produced delay than the variables in quadrants two to four. Variables in quadrant II have a strong impact but this is based on a slow reaction. They can still be suitable for intervention, if the goal can be achieved in a slower manner. Variables situated on the quadrant lines between I and II may also be suitable. Variables in quadrant III are not as favorable, as the impact is low, though achieved rather quickly. The variables in quadrant IV have almost no impact and react slowly, thus they are assumed not to be suitable for intervention implementation* [1, 2].

1. Beck M, Schoenenberger L and Schenker-Wicki A. How managers can deal with complex issues: a semi-quantitative analysis method of causal loop diagrams based on matrices. Zurich: Department of Business Administration - University of Zurich, 2012.
2. Schoenenberger L, Schenker-Wicki A and Beck M. Analysing Terrorism from a Systems Thinking Perspective. *Perspectives on Terrorism*. 2014; 8: 16-36.
